# Silencing SHMT2 inhibits the progression of tongue squamous cell carcinoma through cell cycle regulation

**DOI:** 10.1186/s12935-021-01880-5

**Published:** 2021-04-16

**Authors:** Yan Liao, Fang Wang, Yadong Zhang, Hongshi Cai, Fan Song, Jinsong Hou

**Affiliations:** 1 Department of Oral and Maxillofacial Surgery, Guanghua School of Stomatology, Hospital of Stomatology, Sun Yat-Sen University, Guangzhou, 510055 Guangdong China; 2 Guangdong Provincial Key Laboratory of Stomatology, Sun Yat-Sen University, Guangzhou, 510055 Guangdong China

**Keywords:** SHMT2, Tongue squamous cell carcinoma, Survival analysis, Cell cycle, Weighted gene co-expression network analysis, Gene set enrichment analysis

## Abstract

**Background:**

Serine hydroxymethyltransferase 2 (SHMT2) is a vital metabolic enzyme in one carbon metabolism catalyzing the conversion of serine to glycine, which has been reported to play a crucial role in the progression of tumors. However, its function in tongue squamous cell carcinoma (TSCC) remains unclear.

**Methods:**

SHMT2 expression was analyzed using samples in online databases, and was assessed through immunohistochemistry staining of collected clinical specimens. The correlation between SHMT2 expression and the cell cycle was predicted through bioinformatic analysis, including weighted gene co-expression network analysis (WGCNA) and gene set enrichment analysis (GSEA). After transfection with siRNA, CCK8 assay, Edu staining, flow cytometry, trans-well assay, and wound healing experiments were performed to verify the functional role of SHMT2 in vitro. A stable cell line with SHMT2 silencing was established to detect the oncogenic function of SHMT2 in vivo.

**Results:**

The expression of SHMT2 was up-regulated in TSCC tissues and cell lines compared with normal groups, and highly expressed SHMT2 significantly indicated a poorer clinical outcome for TSCC patients. Bioinformatic analysis found that high expression of SHMT2 was closely related with biologic process including cell cycle and cell cycle G1/S transition. Down regulating of SHMT2 significantly suppressed the proliferation, invasive and migrative ability of TSCC cells, and induced the prolongation of the G1 phase of the cell cycle in vitro. Furthermore, western blot showed that cell cycle-related regulators such as cyclin-dependent kinase 4 (CDK4) and cyclinD1 expression levels were decreased, while the expression levels of the cyclin-dependent kinase inhibitors p21^Cip1^ and p27^Kip1^ were increased after SHMT2 knockdown. Silencing SHMT2 in the HN6 cell line using short hairpin RNA also impeded tumor growth in vivo.

**Conclusions:**

Overexpression of SHMT2 in TSCC indicated low survival rates, and was associated with aggressive behaviors of TSCC. It was also found to be involved in cell cycle regulation of TSCC cells. SHMT2 may serve as a novel prognostic indicator of TSCC.

**Supplementary Information:**

The online version contains supplementary material available at 10.1186/s12935-021-01880-5.

## Introduction

TSCC is one of the most common types of malignant oral squamous cell carcinoma, which is typically characterized by high incidence of lymph node metastasis and distant metastasis due to frequent tongue movement [1]. Many risk factors contribute to the occurrence of TSCC, among which the most significant ones are smoking and chewing betel quid [2]. Although the diagnosis and treatment of TSCC have been improved, the cure rate of TSCC is still not ideal, which seriously affects the life quality of patients such as eating and speech. The high recurrence and lymph node metastasis rate of patients with the condition, even after treatment, are the most difficult problems facing oral clinicians at present [3, 4]. It is therefore necessary to clarify the underlying molecular mechanisms of TSCC and to find a novel tumor biomarker for the diagnosis of early-stage TSCC, in order to provide timely treatment and improve the survival rate of patients.

Tumor cells undergo active metabolism during the cell cycle to support the rapid synthesis of metabolites [5]. In order to meet the energy requirements of rapid cell proliferation, tumor cells reprogram their metabolism to produce ATP through glycolysis rather than through oxidative phosphorylation, a phenomenon which is known as the Warburg effect [6–8].The Warburg effect involves a series of metabolic alterations in tumors, including aerobic glycolysis, abnormally high glucose uptake and one-carbon metabolism [9]. One-carbon metabolism plays a pivotal role in these metabolic adaptions, providing essential precursors for the synthesis of protein, nucleotides and lipids, and methyl groups for the production of DNA [10]. Mitochondrial serine hydroxylmethyltransferase2 (SHMT2) has been found to be a central enzyme in one-carbon metabolism [9].

*SHMT2*, located on human chromosome 17, is responsible for encoding the mitochondrial form of a pyridoxal phosphate-dependent enzyme [11]. An important paralog of SHMT2 is SHMT1, which is mainly present in the cytoplasm [12]. SHMT2 is primarily involved in catalyzing the conversion of serine to glycine, and simultaneously transfers β-carbon from serine to hydrolyzed tetrahydroflate (THF) to form 5,10-methylenettrahydrofolate (Me THF) [11, 13]. As the hub of serine catabolism and single carbon metabolism, SHMT2 plays a regulatory role, in which small molecular metabolites control cell proliferation [14]. Moreover, SHMT2 has been reported to be up-regulated in a variety of cancers such as breast cancer, hepatocellular carcinoma, and lung cancer, and elevated expression of SHMT2 is significantly associated with poor prognosis of patients [15–17]. In glioma cells, SHMT2 has been shown to contribute to cell proliferation in ischemic and hypoxia tumor microenvironments [18]. Taken together, SHMT2 appears to play an essential part in the progression of solid tumors, which encouraged us to explore the role of SHMT2 in TSCC.

This study mainly focused on the potential role of SHMT2 in TSCC aggressive progression. We investigated the relationship between SHMT2 expression and the clinicopathological characteristics of the patients. Furthermore, the correlation between SHMT2 and cell cycle was revealed by bioinformatic analysis. A series of functional experiments in vitro and in vivo were conducted to investigate the impact of SHMT2 on cell cycle, proliferation, invasion, migration and epithelial mesenchymal transition (EMT) of TSCC.

## Materials and methods

### Patients and tissue samples

At the Hospital of Stomatology, Sun Yat-Sen University, we collected 91 oral squamous cell carcinoma (OSCC) tissue samples (including 17 highly differentiated samples, 41 moderately differentiated samples and 33 poorly differentiated samples) and 10 adjacent non-tumor tissue (ANCT) samples between September 2016 and January 2020. Another 9 normal oral tissues were selected from oral cancer tissue chip (HOraC080PG01, Shanghai Outdo Biotech Co, Shanghai, China). The cancer patients were selected based on their clinical and pathological information. Patients who had undergone preoperative radiotherapy or chemotherapy were excluded. We took two identical tissue samples from each patient. One was stored at − 80 ℃ for RNA extraction, and the other was fixed in formalin and embedded in paraffin for immunohistochemical analysis. All patients signed informed consent forms, and the study was approved and supervised by the Institutional Research Ethics Committee of the Hospital of Stomatology, Sun Yat-Sen University.

### Cell lines, cell culture, and transfection

Five human TSCC cell lines including HN6, HSC3, CAL33, SCC15, and SCC25, and a normal oral keratinocyte (NOK) were used in this study. SCC15 and SCC25 were purchased from America Type Culture Collection (ATCC, Manassas, VA, USA), HSC3 was purchased from Japanese Collection of Research Bioresources (JCRB) cell bank (Osaka, Japan) and CAL33 was obtained from Deutsche Sammlung von Mikroorganismen und Zellkulturen (DSMZ, Germany). HN6 was donated by professor Liu (Southern Medical University, Guangdong, China). NOK was provided by J. Silvio Gutkind (NIH, Bethesda, MD, USA). HSC3 and CAL33 cells were cultured in Dulbecco’s modified Eagle’s Serum (DMEM, Gibco, USA) supplemented with 10% fetal bovine serum (FBS, Gibco, USA). HN6, SCC15, and SCC25 cells were grown in DMEM/Hams F12 medium (DMEM/F12, Gibco, USA) containing 10% FBS. NOK cells were grown in keratinocyte SFM medium mixed with growth factor. All cells were cultured at 37 ℃ in a humidified incubator with 5% CO_2._

RNA interference and transfection were conducted to test the function of SHMT2 in TSCC cells. The short-interfering RNA was synthesized by Ruibo Biotechnology Co., Ltd. (Guangzhou, China). The sequences specific to SHMT2 were:

si-SHMT2-1: 5′-GTGATTCCCTCGCCTTTCA-3'.

si-SHMT2-2: 5′-AGACCGAAGTGCCATCACA-3'.

si-SHMT2-3: 5′-CGAGGCTACTCACTGGTAT-3'.

HN6 and HSC3 cells were cultured in six-well plates. After the cell density reached 80%, the cells were washed twice with PBS. The transfection reagent was then prepared. For mixture one,10 μl lipofectamine RNAi-MAX Transfection Reagent (Invitrogen; Thermo Fisher Scientific Inc.) was added to 125 µl Opti-MEM (Invitrogen; Thermo Fisher Scientific Inc.) medium according to the manufacturer’s instructions. For mixture two, 10 μl siRNA was diluted with 125 µl Opti-MEM. Mixture one and mixture two were mixed in one tube and incubated for 10 min at room temperature. After incubation, cells were grown with 250 µl of the mixture and 750 µl complete medium at 37 ℃ with 5% CO_2_ for 12 h. Then 1 ml of complete medium was introduced into the transfection medium. Cells were collected 48 h after transfection for subsequent experiments.

### Immunohistochemistry

Immunohistochemistry (IHC) was performed as follows. The paraffin-embedded tissues were cut into 4.0 μm sections for dewaxing and dehydration. Staining was carried out using antigenic retrieval and goat serum block (AR0009, Boster Bio, Wuhan, China). The sections were incubated with primary antibody against SHMT2 (1:500, GTX125939, GeneTex), Ki-67 (1:200, ab16667, Abcam), p21^Cip1^ (1:50, 12D1, Cell Signaling), p27^Kip1^ (1:50, ab32034, Abcam), and CyclinD1 (1:50, E3P5S, Cell Signaling), CDK4 (1:500, D9G3E, Cell Signaling) at the recommended dilution overnight at 4 ℃. Staining was then developed using diaminobenzidime (DAB, GK600510, Gene Tech, China), and the cell nuclei were stained with hematoxylin (D006, Nanjing, Jiancheng, Biotech, China). The staining score was evaluated by multiplying the staining intensity and the percentage of positive cells. The intensity of staining was scored as 0 = negative, 1 = weakly positive, 2 = positive, 3 = strongly positive. The percentage of positive cells were defined as 0% = 0; < 10% = 1; ≥ 10 to < 50% = 2; ≥ 50% to < 75% = 3; ≥ 75% = 4. The total IHC scores ranged from 0 to 12 and tissues with a score ≥ 6 were assigned to the high expression group, while those with scores < 6 were placed in the low expression group.

### Western blot analysis

Cells were lysed in RIPA buffer (CW2333S, CWBIO, China) supplemented with protease inhibitor cocktail set 1 (539131, Millipore, USA) and phosphatase inhibitor. Protein concentrations were measured using BCA protein assay kits (CW0014S, CWBIO). Equal amounts of total cell lysate were separated using 10% SDS-PAEG (P0014B, CWBIO) and transferred to PVDF membranes (ISEQ00010, Millipore, USA) blocked with 5% bovine serum albumin (0332, Ameresco) for 1 h at room temperature. Then the blots were incubated with the primary antibodies against SHMT2 (1:1,000, GTX125939, GeneTex), E-cadherin (1:1,000, 24E10, Cell Signaling), ZO1 (1:1000, D7D12, Cell Signaling), N-cadherin (1:1,000, D4R1H, Cell Signaling), Vimentin (1:1,000,D21H3,Cell Signaling, β-catenin (1:1,000, D10A8, Cell Signaling), p21^Cip1^ (1:1000, 12D1, Cell Signaling), p27^Kip1^ (1:1000, D69C12, Cell Signaling), Cyclin D1(1:1000, E3P5S, Cell Signaling), CDK4(1:1000, D9G3E, Cell Signaling), β-actin (1:1000,13E5, Cell Signaling), and GAPAH (1:1,000,D16H11, Cell Signaling) at 4 ℃ overnight. Membranes were incubated in HRP-conjugated secondary antibody (1:2000, 7074S, Cell Signaling) for 1 h at room temperature. The protein bands were exposed using a chemiluminescent substrate (WBKLS0500, Millipore, USA), and quantitatively analyzed using ImageJ [19].

### Quantitative reverse transcription polymerase chain reaction (qRT-PCR)

Total RNA was extracted from the cultured cells and tissues using RNAzol® RT (RN190, Molecular Research Center, USA) according to the manufacturer’s protocol. The quality and quantity of the RNA were examined using a NanoDrop® ND-1000 Spectrophotometer (Thermal Fisher, Wilmington, DE, USA). RNA was reverse transcribed into cDNA using the PrimeScript RT reagent Kits (RR036A, Takara Bio, Japan). All samples were tested using SYBR Master Mix (11201ES208, Yeasen, China). The quantity of mRNA was calculated using method 2^−ΔΔCt^ and normalized to GAPDH. The prime sequences were as follows:

SHMT2-forward: 5′-GCCACGGCTCATCATAGCTG-3'.

SHMT2-reverse: 5′-AGCAGGTGTGCTTTGACTTCA-3'.

CDKN1A-forward: 5′-GTCCACTGGGCCGAAGAG-3'.

CDKN1A-reverse: 5′-TGCGTTCACAGGTGTTTCTG-3'.

CDKN1B-forward: 5′-TTCATCAAGCAGTGATGTATCTGA-3'.

CDKN1B-reverse: 5′-AAGAAGCCTGGCCTCAGAAG-3'.

CCDN1-forward: 5′-AACTACCTGGACCGCTTCCT-3'.

CCDN1-reverse: 5′-CCACTTGAGCTTGTTCACCA-3'.

CDK4-forward: 5′-GTCGGCTTCAGAGTTTCCAC-3'.

CDK4-reverse: 5′-TGCAGTCCACATATGCAACA-3'.

GAPDH-forward: 5′-CCTTCCGTGTCCCCACT-3'.

GAPDH-reverse: 5′-GCCTGCTTCACCACCTTC-3'.

### CCK-8 assay

The cell viability was detected using cell counting Kit-8 s (CCK-8, 40203ES80, Yeasen, China), at 0, 24, 48, 72, 96 h. In brief, 2 × 10^3^(HN6) or 3 × 10^3^ (HSC3) cells/well were seeded in 96-well culture plates, in triplicate. At the end of the experiment, 100 μl of 10% CCK-8 reagent (10 μl CCK-8 and 90 μl serum-free media) was added to each well, and the plates were incubated for 1 h at 37℃. Absorbance was measured at 450 nm using a microplate reader (Bio-Read, USA).

### Migration and invasion assays

Cell motility was evaluated by migration and invasion assays using Matrigel (10 mg/ml, 354,234, Corning, China) and trans-well filters (pore size, 8 µm; BD Biosciences). And for invasion assay, 0.33 mg/ml Matrigel was coated on the upper chamber of Trans-well assay plates 2 h in advance. While for migration assay, Matrigel was not required. Briefly, 3 × 10^4^ HN6 cells or 4 × 10^4^ HSC3 cells were seeded into the upper chambers with 200 µl serum-free medium, while the lower chambers were supplemented with 600 µl complete medium. After 24 h, the cells on the upper filter were removed, while those on the lower chamber were fixed with paraformaldehyde and stained with 0.1% crystal violet. The cells in five random fields were subsequently counted under the microscope to assess the number of nuclei.

### Wound healing assay

Cell migration and repair ability were assessed using wound healing assays. HN6 and HSC3 cells were seeded in six-well plates and cultured to cover 90% of each well. Scratches in the cell layer were made with a 1 ml sterile pipette tip, and cells were washed twice with phosphate-buffered saline (PBS). After that, cells were cultivated with serum-free medium. Cells were photographed under a light microscope at 0 h, 24 h, and 48 h. The ImageJ software was used to quantify the distance between cell edges, and to calculate the wound healing rate.

### Flow cytometry analysis

Following centrifugation, 2 × 10^5^–2 × 10^6^ cells were collected, and the supernatant removed. The cells were resuspended in PBS, centrifuged again, and the supernatant discarded. Then 1 ml DNA staining solution and 10 µl permeabilization solution (Multi Sciences) was added to each tube, and cells were incubated at room temperature without light. Finally, cells were measured using Beckman flow cytometry and data was analyzed using FlowJo (https://www.flowjo.com/) Software.

### EDU staining

KFluor488 Click-it EdU imaging detection kits (KeyGen Biotech, Jiangsu, China) were used to assess the cell proliferation**.** Cells were grown in 24-well plates, and were incubated with 50 uM EdU for 2 h at temperature. Cells were fixed with 4% paraformaldehyde in PBS and osmosis was promoted with 0.5% Triton X-100 in PBS. Click-iT reaction mixture was prepared for EdU detection, and Hoechst 33,342 was used to re-stain DNA. Finally, the cells were photographed with an inverted fluorescence microscope and counted using ImageJ software, based on the stained nuclei.

### Lentiviral short hairpin RNA (shRNA) transfection

A stable knockdown of SHMT2 expression using shRNA was established, to detect the function of SHMT2 in vivo. The lentivirus packaging process was carried out by Cyagen US Inc. Lentivirus carrying shRNA targeting SHMT2 was used to achieve stable knockdown of SHMT2 expression. The sequence of the targeting vector was:

HN6-shSHMT2-1: 5′-CTTCGAGTCTATGCCCTATAACTCGAGTTATAGGGCATAGACTCGAAG -3′

HSC3-shSHMT2-2: 5′-CGGAGAGTTGTGGACTTTATACTCGAGTATAAAGTCCACAACTCTCCG -3′

### Tumor xenografts

Female 5-to 6-week-old NOD/SCID mice were randomly divided into two groups, the sh-SHMT2 group and the control group, with eight mice in each group. Then, 2 × 10^6^ HN6 cells suspended in 200 μl PBS were subcutaneously inoculated into the left inner thighs of mice. Transplanted tumors were observed weekly, and the tumor xenografts were harvested, photographed, and fixed for the IHC four weeks after injection. Tumor volume was calculated using the following formula: volume = (width^2^ × length)/2. All animal experiments were approved by Sun Yat-Sen University’s Animal Experiment Ethics Committee and conducted in accordance with their principles of animal welfare.

### Data collection and analysis

OSCC samples (313) and normal oral mucosal tissue samples (30) were selected from the head and neck squamous cell carcinoma datasets in the Cancer Genome Atlas (TCGA) database (https://www.cancer.gov/tcga), according to the OSCC classification criteria. Carcinomas originated from the tongue, floor of the month, buccal mucosa, hard palate, alveolar ridge, and oral cavity. Samples with complete clinical information were selected for further analysis. The 313 OSCC samples selected were divided into a SHMT2 low-level group and a high-level group, according to the median expression level of SHMT2. The R software (http://www.R-project.org/) was used to perform survival analysis, weighted gene co-expressed network analysis (WGCNA) and gene set enrichment analysis (GSEA). The results of the bioinformatic analysis of the TCGA database were validated using GSE30784 from the Gene Expression Omnibus database (https://www.ncbi.nlm.nih.gov/).

### Weighted gene co-expressed network analysis

The top 50% genes with the highest variance were selected among 10,215 genes for WGCNA analysis using the R “WGCNA” package. A gene expression similarity matrix was constructed with which to analyze the cooperative expression between genes. An adjacency matrix was built based on the above matrix, and an appropriate soft threshold was introduced to establish a scale free network. A topological overlap matrix (TOM) was constructed by defining a measure of node dissimilarity. Network modules were then identified using dynamic hierarchical tree clustering. Finally, we selected the most relevant module with which to further analyze the correlation between modules and clinical parameters in OSCC using Gene Ontology annotation (GO) [20, 21] and the Kyoto Encyclopedia of Genes and Genomes pathway (KEGG) [22] enrichment analysis.

### Gene set enrichment analysis

GSEA software (http://software.broadinstitute.org/gsea/index.jsp) was used to conduct the enrichment analysis on the groups with high expression of SHMT2 and low expression of SHMT2 to evaluate the trend of distribution of SHMT2 in the gene table of OSCC biological phenotypic relevance sequencing, and to determine its contribution to the phenotype. A *P*-value < 0.05 and a false discovery rate (FDR) < 0.05 were considered as credible enrichment.

### Statistical analysis

All in vitro experiments were repeated at least three times. The mean and the standard deviation of data were analyzed by GraphPad Prism 8.0 software. The difference between the two groups was analyzed using two-tailed Student’s *t*-tests, and one-way analysis of variance (ANOVA) were used for the multiple groups. The correlations between SHMT2 expression and the clinicopathological features of the OSCC patients were analyzed using chi-square tests or Fisher’s exact tests. Survival curves were constructed using the Kaplan–Meier method and tested with log-rank. SPSS 25.0 was applied to establish a Cox proportional hazard regression model for univariate and multivariate analysis. The differences were considered statistically significant at *p* < 0.05 (* *p* < 0.05, ** *p* < 0.01, *** *p* < 0.001).

## Results

### SHMT2 is up-regulated in TSCC cells and is associated with poor prognosis for patients

**Fig. 1:**
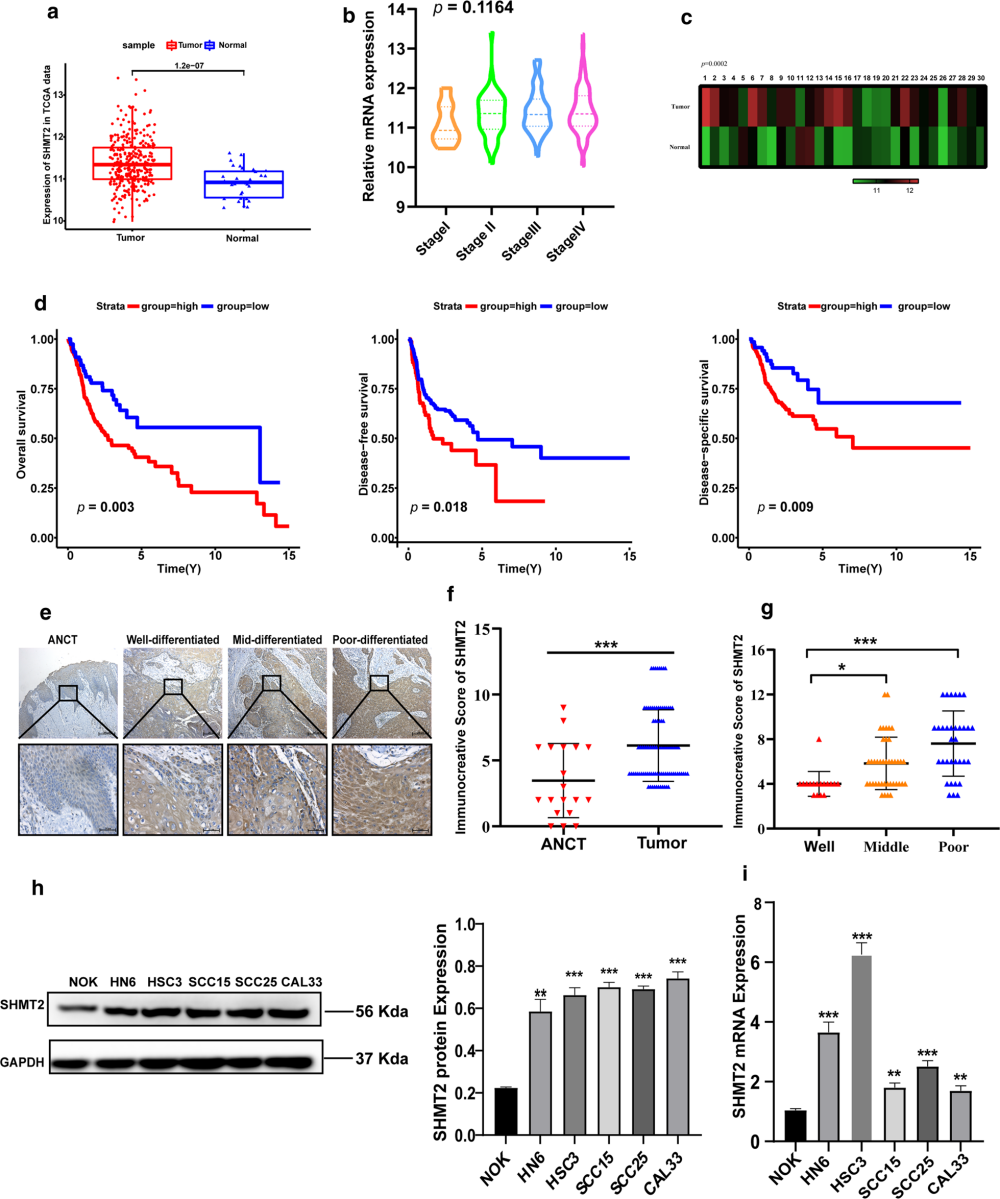
SHMT2 was overexpressed in OSCC tissues and predicted poor prognosis. **a** Relative analysis of *SHMT2* mRNA in OSCC (n = 313) and adjacent normal tissues (n = 30) from the TCGA database. **b** Analysis of *SHMT2* mRNA level in different pathologic stages of OSCC samples in the TCAG database (stage I, n = 19; stage II, n = 54; stage III, n = 56; stage IV, n = 162). **c** SHMT2 expression level in 30 paired oral tumor tissues and adjacent normal tissues in the TCGA database. **d** Kaplan–Meier survival curves for overall survival (n = 312), disease free survival (n = 312) and disease specific survival (n = 295) in OSCC patients with high and low expression of SHMT2. **e** Immunohistochemistry staining of SHMT2 in ANCT and tumors with different degrees of differentiation. Magnification of 100 × (up) and 400 × (down). **f** Immunohistochemical staining score of SHMT2 in ANCT (n = 19) and OSCC (n = 91) tissues. **g** Immunohistochemical staining score of SHMT2 in three different differentiation degrees of OSCC (Well: n = 17, middle: n = 41, poor: n = 33). **h** Western blot pictures and quantitative analysis of SHMT2 in NOK and TSCC cell lines. **i** Real time PCR analysis of *SHMT2* in NOK and TSCC cell lines. *P*-values were obtained using Student's *t*-tests, one-way ANOVA tests, and log-rank tests. All data are shown as mean ± SD. * *p* < 0.05, ** *p* < 0.01, *** *p* < 0.001. OSCC, oral squamous cell carcinoma; TSCC, tongue squamous cell carcinoma ANCT, adjacent noncancerous tissue samples; NOK, normal oral keratinocyte

SHMT2 expression was firstly analyzed among 343 samples from the TCGA database, we found that SHMT2 was significantly up-regulated in 313 oral squamous cell carcinoma (OSCC) samples, when compared with 30 normal tissue samples (*P* < 0.0001) (Fig. 1a). Then, 22 OSCC samples without pathological staging information were deleted and the remaining 291 samples were used to explore the relationship between SHMT2 expression and pathological staging. The results showed that SHMT2 level in the advanced pathological stage of OSCC tissues tended to be higher than that in the early stage, although it was not statistically significant, which may be related to the limited sample size (*P* = 0.1164) (Fig. 1b). To exclude individual differences, we analyzed 30 pairs of OSCC tissues and normal tissues from the TCGA database, and obtained similar results (*P* = 0.0002) (Fig. 1c). Survival analysis showed that high expression of SHMT2 was statistically significant, with lower overall survival rate (*P* = 0.003), shorter disease free survival (*P* = 0.018), and worse disease specific survival (*P* = 0.009) in OSCC patients (Fig. 1d).

We examined our clinical samples for SHMT2 expression in OSCC tissues. Immunohistochemistry staining analysis showed that SHMT2 was overexpressed in tumor tissues compared with adjacent noncancerous tissue samples (ANCT) (Fig. 1e, f). Moreover, the staining scores of moderately and poorly differentiated tumors were higher than those of highly differentiated tumors (Fig. 1e, g). Then, 96 OSCC samples with complete clinical information were selected from the TCGA database to analyze the correlation between SHMT2 and clinicopathological characteristics. Analysis of the relationships among a series of clinicopathological features revealed that expression of SHMT2 was significantly associated with age (*P* = 0.03), pathologic tumor stage (*P* = 0.016), pathologic lymph node metastasis (*P* = 0.039) and clinical stage (*P* = 0.004), alcohol history (*P* = 0.005) and margin status (*P* = 0.045). But there was no significant difference in the expression of SHMT2 with gender, smoking, pathologic metastasis, differential degree, lymph node neck dissection, lymph vascular invasion, perineural invasion and recurrence (Table 1). Furthermore, we selected several important factors concerned by clinicians such as age, gender, clinic T/N/M stage, clinic stage, histologic grade and SHMT2 expression level to conduct univariate and multivariate analysis. A total of 303 OSCC samples were included after the exclusion of incomplete clinical data. Univariate analysis suggested that SHMT2 expression and lymph node metastasis were risk factors for overall survive of oral cancer patients. The result of multivariate analysis demonstrated that SHMT2, lymph node metastasis and differential degree could be independent prognostic factors affecting overall survive time of oral cancer patients (Table 2).

**Table 1:** Correlations between SHMT2 and clinicopathological characteristics of oral squamous cell carcinoma in TCGA database (n = 96)

Characteristics	SHMT2 expression		
	High	Low	*p* value
Anatomic site			
Alveolar Ridge	5	5	0.215
Buccal Mucosa	6	3	
Floor of mouth	20	9	
Hard palate	0	2	
Oral Cavity	11	7	
Tongue	12	16	
Age (years)			
< 61	24	28	0.03*
≥ 61	30	14	
Gender			
Male	36	30	0.618
Female	18	12	
Alcohol history			
Yes	32	36	0.005**
No	22	6	
Tobacco smoking history			
Yes	36	30	0.618
No	18	12	
pT stage			
T1–2	13	20	0.016*
T3–4	41	22	
pN stage			
N^−^	17	20	0.039*
N^+^	37	22	
pM stage			
M^−^	39	34	0.320
M^+^	15	8	
Clinical stage			
I–II	8	17	0.004**
III–IV	46	25	
Histologic grade			
Well	4	10	0.078
Moderately	39	25	
Poorly	11	7	
Lymph node neck dissection			
Yes	52	40	1.000
No	2	2	
Lymph vascular invasion			
Yes	20	10	0.165
No	34	32	
Perineural invasion			
Yes	26	23	0.520
No	28	19	
Close or positive margin			
Yes	22	9	0.045*
No	32	33	
Recurrence			
Yes	21	10	0.117
No	33	32	

pT stage pathologic T stage, pN stage pathologic N stage, pM stage pathologic M stage

^*^represents *P* < 0.05, **represents *P* < 0.01, ***represents *P* < 0.001

**Table 2:** Univariate and multivariate analysis of various clinicopathologic characteristics related with overall survival in OSCC patients(n = 303)

	Univariate analysis	Multivariate analysis		P
	HR (95% CI)	P	HR (95% CI)	
Gender	0.924 (0.656–1.300)	0.648		
Age	1.185 (0.853–1.646)	0.311		
cT stage	1.344 (0.951–1.899)	0.094		
cN stage	1.471 (1.060–2.039)	0.021*	1.534 (1.008–2.335)	0.046*
cM stage	0.692 (0.171–2.799)	0.605		
Clinical stage	1.342 (0.923–1.950)	0.123		
Histological grade	1.282 (0.990–1.661)	0.06	1.317 (1.011–1.715)	0.041*
SHMT2 level	1.522 (1.094–2.117)	0.013*	1.452 (1.034–2.037)	0.031*

cT stage clinic T stage, cN stage clinic N stage, cM stage clinic M stage

^*^represents *P* < 0.05, **represents *P* < 0.01, ***represents *P* < 0.001

To investigate SHMT2 expression in TSCC cells, we tested the mRNA and protein levels of SHMT2 in five TSCC cell lines (HN6, HSC3, CAL33, SCC15, and SCC25) and in one normal oral keratinocyte (NOK). Both mRNA and protein expression of SHMT2 in oral cancer cells were higher than that in normal oral keratinocyte (Fig. 1h, i). These results indicated that high expression of SHMT2 in TSCC might be indicative of an unfavorable prognosis.

### Construction of a weighted co-expression network and identification of the key module

**Fig. 2:**
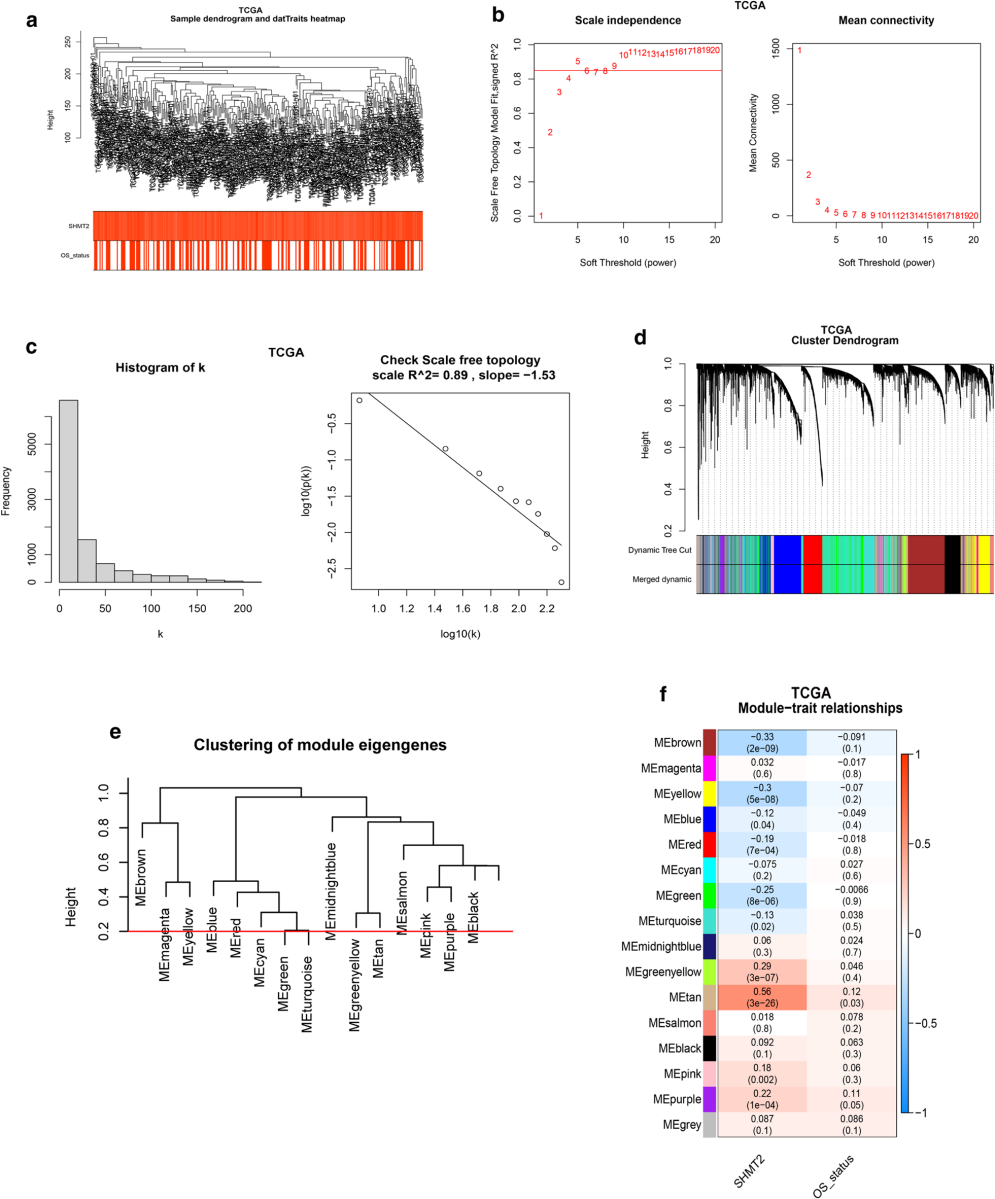
Construction of weighted co-expression network and identification of the key module. **a** Hierarchical clustering dendrogram of samples in TCGA database (n = 306). **b** Analysis of the scale free fit index and the mean connectivity for various soft threshold power. **c** Scale free topology test based on β = 5. **d** Cluster dendrogram of genes with dissimilarity based on topological overlap. Branches of the clustering tree represent merged modules, and different colors correspond to different modules. **e** Cluster dendrogram of module eigengenes: modules below the red line (0.2) are merged. **f** Correlation coefficient and *p*-value for module-trait relationships. Each row is a module eigengene

WGCNA is a typical systems biology algorithm for constructing gene co-expression networks, which aims to find gene network modules of cooperative expression and explore the correlation between gene networks and clinical parameters. In this study, seven abnormal cases were excluded, according to the connection filtering threshold and a total of 306 samples were clustered using WGCNA (Fig. 2a). Based on a function in the WGCNA package, we chose the power of β = 5 (scale free R^2^ = 0.84) as the soft-thresholding to satisfy the scale free topology (Fig. 2b, c). The adjacency matrix was then converted into a topological matrix (TOM) by calculating the topological overlap between genes. As shown in the clustering dendrogram, 15 modules were merged, based on the height of module eigengenes higher than 0.2 (Fig. 2d, e). Among these modules, the tan module (*r* = 0.56, *P* < 0.0001) had the closest correlation with SHMT2. The tan module was therefore chosen as the key module for subsequent analysis (Fig. 2f). We also conducted WGCNA for GSE30784 to validate the results of OSCC samples in the TCGA database (Additional file 1: Fig. S1).

### Go, KEGG, and GSEA analysis of the hub module

**Fig. 3:**
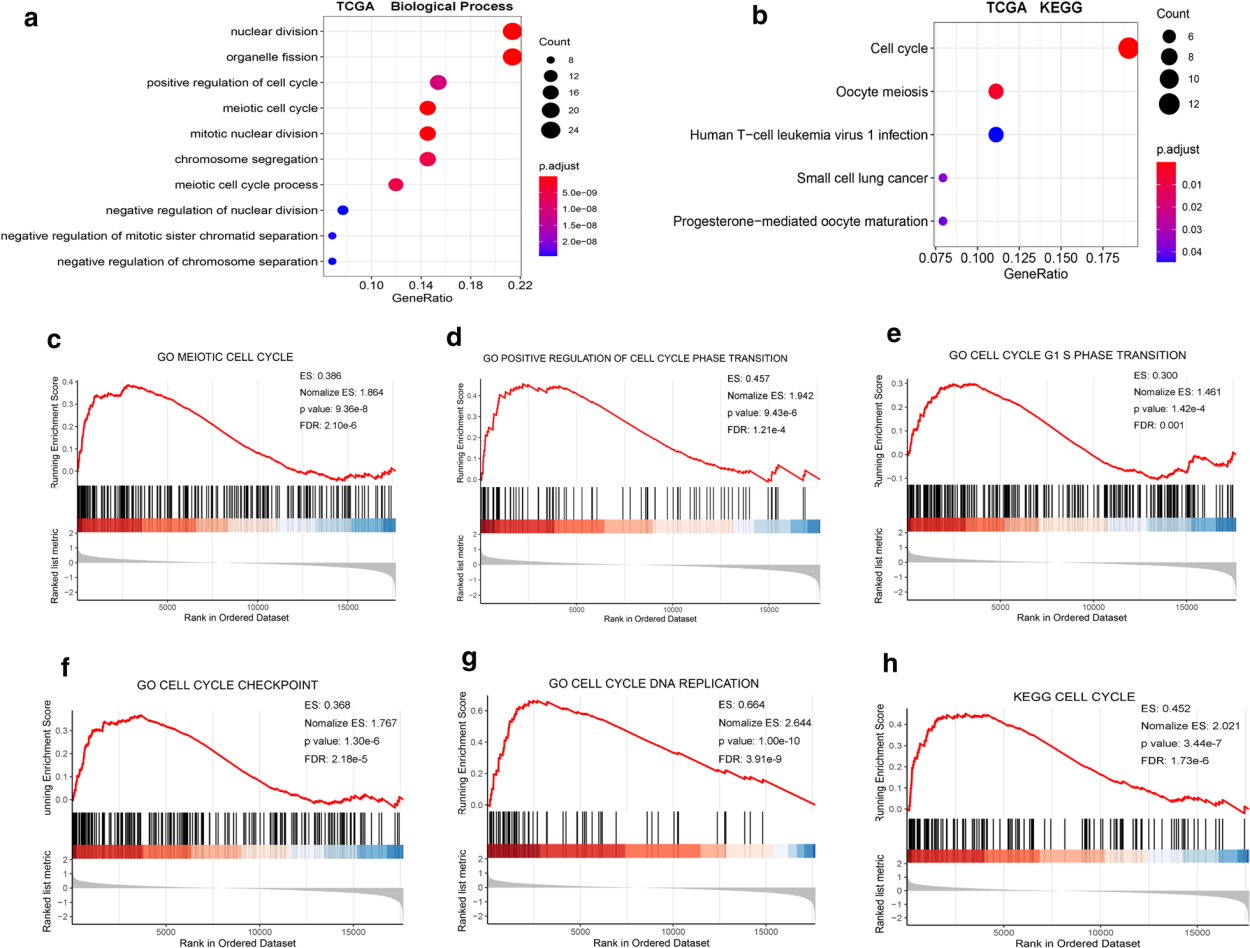
SHMT2 was positively involved in cell cycle regulation of TSCC according to GO, KEGG and GSEA. **a** Biological process analysis for the key module containing SHMT2. **b** KEGG enrichment analysis for the key module containing SHMT2. **c**–**h** GSEA enrichment analysis: GO and KEGG pathway genes in SHMT2 high versus low samples in TCGA database. ES, enrichment score

We performed GO annotation and KEGG enrichment analysis for the tan module, to explore the biological function of SHMT2, using the R package “cluster Profiler”. The results of the GO analysis indicated that the tan module was mainly involved in the biological processes of positive regulation of cell cycle and meiotic cell cycle (Fig. 3a). KEGG analysis provided similar results, with the genes being most significantly enriched in the cell cycle (Fig. 3b).

GSEA was used to validate the relationship between SHMT2 expression and pathways, using the GSEA software. The terms meiotic cell cycle, positive regulation of cell cycle transition, cell cycle G1/S phase transition, cell cycle check point and cell cycle DNA replication were significantly enriched (Fig. 3c-h). These results were confirmed using the GSE30784 dataset (Additional file 1: Fig. S2).

### SHMT2 knockdown inhibits TSCC cell proliferation, inducing cell cycle arrest in G1 phase

**Fig. 4:**
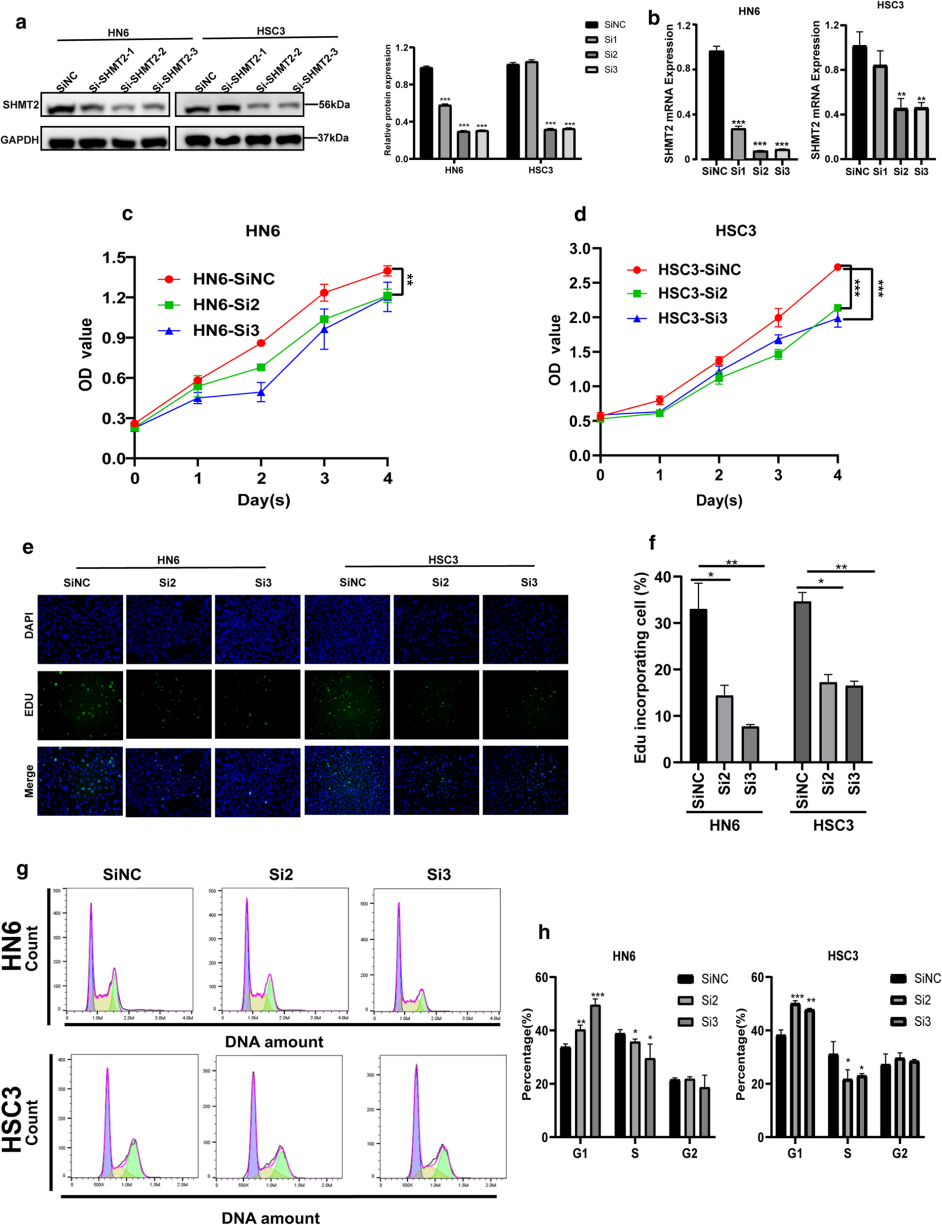

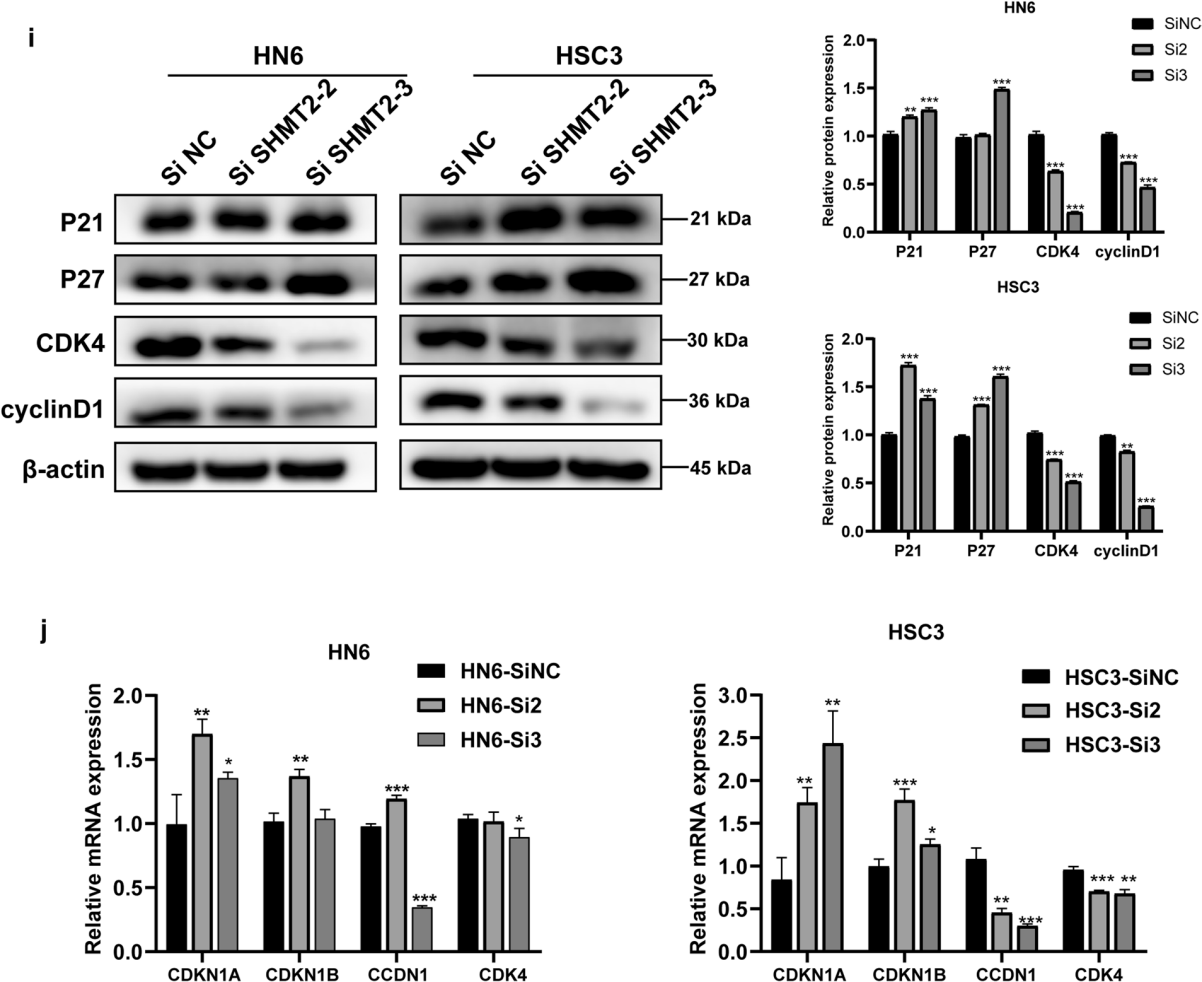
Knockdown of SHMT2 inhibited the proliferation and modulates the TSCC’s cell cycle in vitro. **a**, **b** Western blot analysis and real time PCR of HN6 and HSC3 cells transfected with SHMT2 siRNA. **c**, **d** CCK8 assay performed on HN6 and HSC3 cells after transfection to determine the cell viability. **e**, **f** Representative images of and quantification of EdU stained cells in negative control and SHMT2 siRNA transfected groups. **g**, **h** Flow cytometric analysis of indicated cells, representative graph (left) and quantitative analysis (right). **i** Western blot analysis of several cell cycle regulators including P21, P27, CDK4, and cyclin D1 expression in indicated cells. **j** Real time PCR analysis of several cell cycle regulators including *CDKN1A*, *CDKN1B*, *CCDN1* and *CDK4* in HN6 and HSC3. *P*-values were obtained using Student's *t*-tests and two-way ANOVA tests * *p* < 0.05, ** *p* < 0.01, *** *p* < 0.001

Considering the pivotal role of SHMT2 in cell cycle regulation, si-RNA specifically designed for *SHMT2* was transfected into HN6 and HSC3 cell lines to verify its functional role in vitro. The knockdown effect of SHMT2 was detected by PCR and Western blotting. The results showed that the mRNA and protein levels of SHMT2 were significantly down-regulated, with the down-regulation efficiency reaching about 70% (Fig. 4a, b). We selected two sequences with a good silencing effect, si-SHMT2-2 and si-SHMT2-3, for subsequent experiments. CCK-8 assay results demonstrated that SHMT2 knockdown suppressed the cell viability of HN6 and HSC3 cell lines compared with the control group (Fig. 4c, d). We conducted 5-ethynyl-2′-deoxyuridine (EdU) staining to assess OSCC cell proliferation (Fig. 4e). Images of EdU staining showed that SHMT2 down-regulation significantly reduced the proportion of EdU stained cells (Fig. 4f). To further explore the mechanism by which SHMT2 affects TSCC cell proliferation, we evaluated the cell cycle alternation after siRNA treatment. Flow cytometry analysis showed that the proportion of cells blocked in G1 phase of SHMT2 knockdown group was significantly higher than that of the control group, whereas the number of cells in S phase of si-SHMT2 treated were decreased as compared with control group cells (Fig. 4g, h). We then examined changes in several cell cycle regulators correlated with the G1/S transition through RT-qPCR and western blotting after SHMT2 knockdown. As shown in western blot analyze, the protein levels of the cell cycle inhibitors p21^Cip1^ and p27^Kip1^ increased by SHMT2 knockdown in HN6 and HSC3. Conversely, the protein levels of the cell cycle promoters cyclinD1 and CDK4 decreased in SHMT2 silenced cells (Fig. 4i). Consistently, RT-qPCR results revealed that after si-SHMT2 transfection, the mRNA level of *CDKN1A* and *CDKN1B* were upregulated, while the mRNA level of *CCDN1* and *CDK4* were downregulated in HN6 and HSC3 (Fig. 4j).

### SHMT2 knockdown inhibited the invasive and migrative ability of TSCC cells

**Fig. 5:**
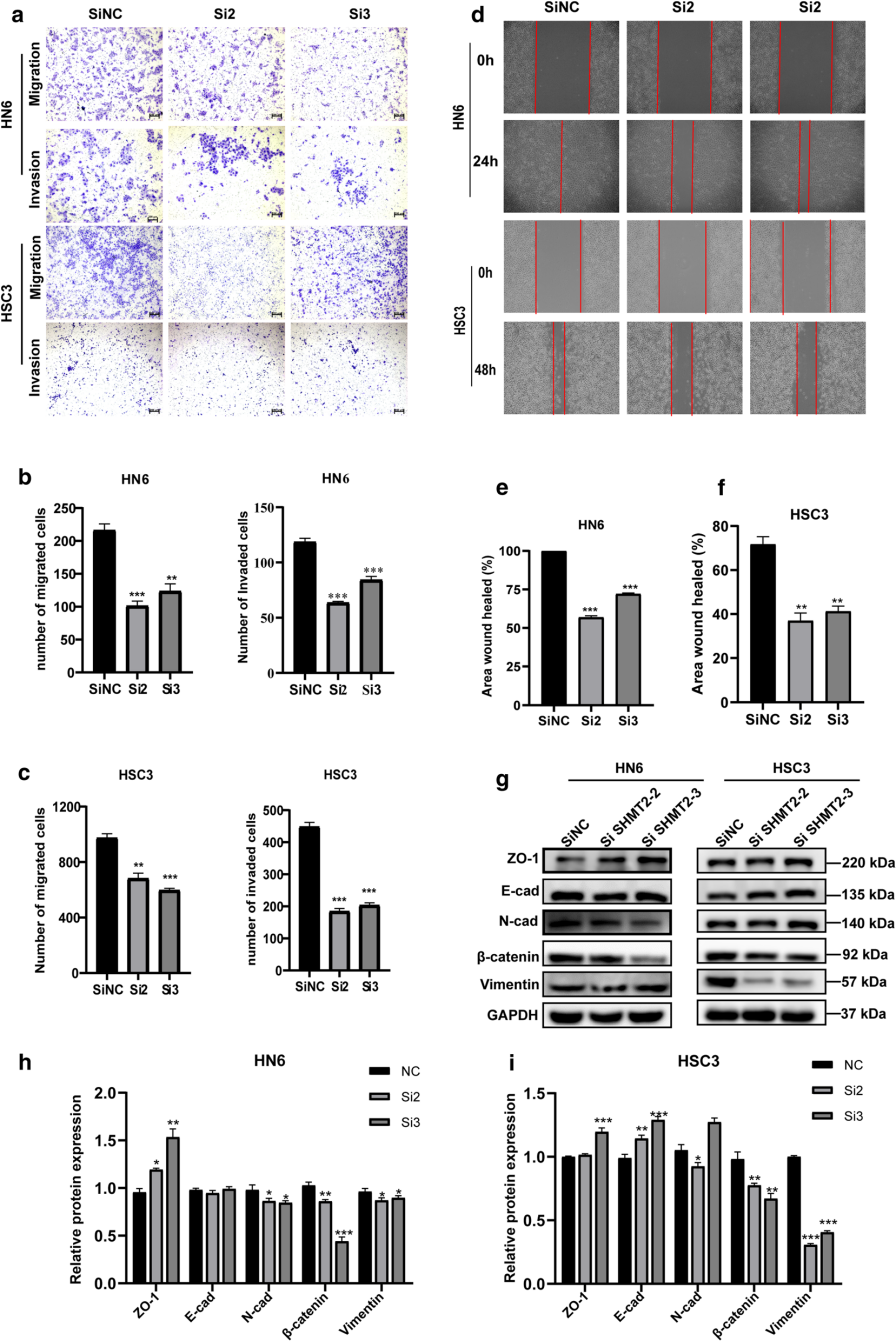
Knockdown of SHMT2 impaired TSCC invasive, migrative and epithelial mesenchymal transition ability. **a** Representative photograph of trans-well assays in HN6 and HSC3 cells after transfection with si-RNA. **b**, **c** Quantitative analysis of trans-well assays in HN6 and HSC3. **d** Wound healing assay images of HN6 and HSC3 cells after SHMT2 down-regulation. **e**, **f** Quantitative analysis of wound healing assay in HN6 and HSC3. **g** Western blot analysis images of EMT-related proteins in HN6 and HSC3 cells after SHMT2 knockdown. **h**, **i** Quantitative analysis of EMT-related proteins in HN6 and HSC3 transfected with si-RNA. *P*-values were obtained by Student's *t*-tests, * *p* < 0.05, ** *p* < 0.01, *** p < 0.001. EMT, epithelial mesenchymal transition; E-cad, E-cadherin; N-cad, N-cadherin

To further examine the functional role of SHMT2, we carried out trans-well and wound healing experiments. It was found that the number of migrating and invasive cells were decreased in the si-SHMT2 treated group when compared with the corresponding control group in HN6 and HSC3 (Fig. 5a–c). The results of the wound healing experiments revealed that down-regulation of SHMT2 reduced the healing area of HN6 and HSC3 cells (Fig. 5d–f). Western blotting was performed to measure changes in epithelial mesenchymal transition-related proteins level (Fig. 5g). We found that mesenchymal phenotype markers including N-cadherin, β-catenin, and Vimentin were downregulated, whereas epithelial phenotype markers such as ZO-1 and E-cadherin were up-regulated after SHMT2 knockdown (Fig. 5h, i). Unexpectedly, compared with control group, E-cad expression in HN6 treated cells showed negligible change and N-cad level in si-SHMT2-3 of HSC3 cells increased abnormally, which may be led by complex protein expression regulatory network. These results indicated that SHMT2 exerted an impact on aggressive behaviors of TSCC cells.

### SHMT2 silencing inhibits cellular proliferation, invasion, migration and tumor formation

**Fig. 6:**
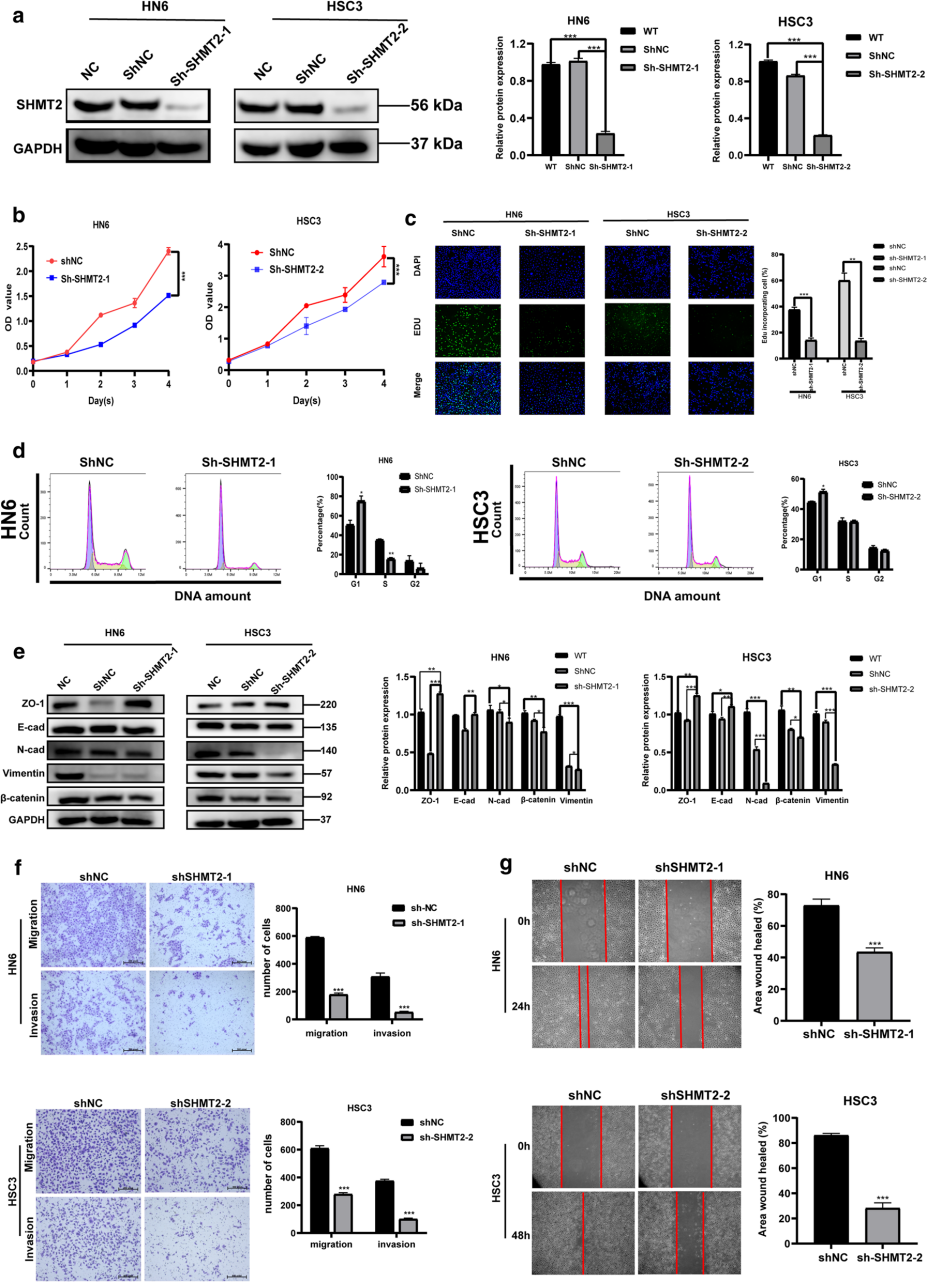

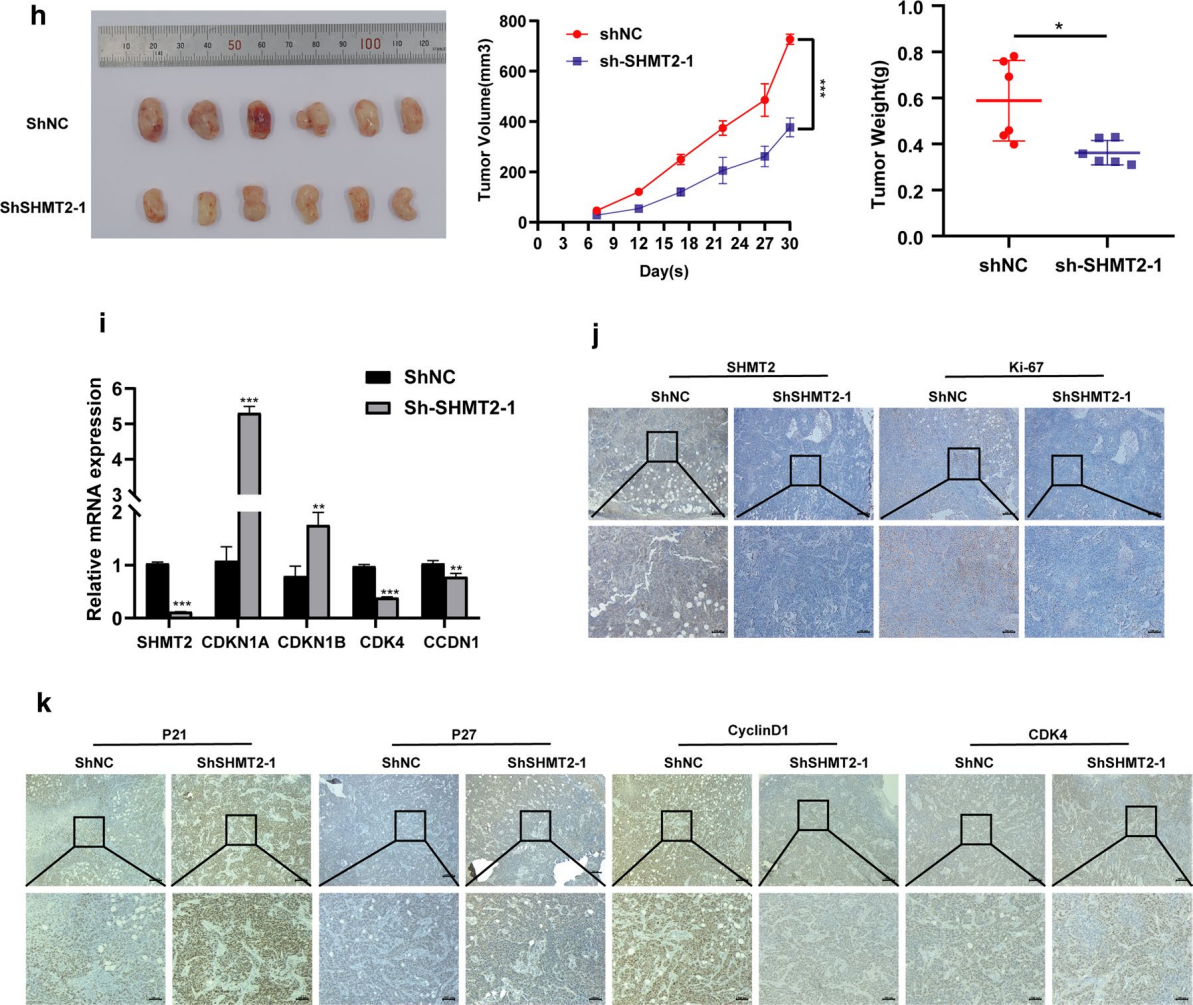
Silencing SHMT2 inhibited the invasive, migrate ability and EMT, and suppressed TSCC cell growth in vitro and in vivo. **a** Western blot analysis and quantitative calculation for SHMT2 level in HN6 and HSC3 cells after lentiviral transfection. **b** Cell viability for HN6 and HSC3 were detected by CCK8 assay. **c** Images of EdU staining in sh-SHMT2 and sh-NC group (left), and quantitative analysis of EdU stained cells in HN6 and HSC3 (right). **d** Flow cytometric analysis of sh-RNA treated cells. **e** Western blot analysis confirmed that silenced SHMT2 expression inhibited EMT of TSCC cells. **f** Representative photos of trans-well assay in HN6 and HSC3 cells after treated with shRNA (left), and quantification of cell numbers in HN6 and HSC3 (right). **g** Representative photos of wound healing assay in HN6 and HSC3 cells after treated with shRNA (left), and quantification of cell numbers in HN6 and HSC3 (right). **h** Image for tumors taken from mice injected with sh-NC and sh-SHMT2 HN6 cells; Tumor volume growth curve of mice; Tumor weight was measured after mice were sacrificed. **i** Real time PCR was used to evaluate expression of *SHMT2*, *CDKN1A*, *CDKN1B*, *CCDN1* and *CDK4* in mouse tumors. **j**, **k** Immunohistochemistry staining was performed to analyze protein expression of SHMT2, Ki-67, P21, P27, cyclinD1, and CDK4 in tumor tissue specimens from sh-SHTMT2 and control groups of mice. Magnification at 50 × (up) and 100 × (down). *P*-values were obtained by Student's *t*-tests and two-way ANOVA tests * *p* < 0.05, ** *p* < 0.01, *** *p* < 0.001. Sh, short hairpin RNA

To further investigate the oncogenic function of SHMT2 in vivo, we constructed a lentivirus knockdown-stable strain targeting *SHMT2*. Two different stable strain sequences, sh-SHMT2-1 and sh-SHMT2-2 were transfected into HN6 cells and HSC3 cells, respectively. Western blotting verified that the two different sequences of sh-RNA significantly reduced the expression level of SHMT2 when compared with sh-NC group (Fig. 6a). The results of CCK-8 assays showed that silencing SHMT2 could inhibit the viability of OSCC cells (Fig. 6b). EdU staining showed that the proportion of EdU stained cells were significantly reduced by SHMT2 silencing (Fig. 6c). Flow cytometry analysis revealed that cell cycle of sh-SHMT2 cells were significantly blocked in G1 phase compared with sh-NC group (Fig. 6d). Western blot analysis of the expression of EMT-associated proteins indicated that SHMT2 silencing in HN6 and HSC3 significantly down-regulated mesenchymal phenotypic markers such as N-cadherin, β-catenin and Vimentin, while epithelial phenotypic markers including ZO-1, E-cadherin were significantly up-regulated (Fig. 6e). It was found that the number of migrating and invasive cells also differed in the sh-SHMT2 treated group and the sh-NC group. SHMT2 silencing significantly inhibited migration and invasion of TSCC cells (Fig. 6f). Wound healing results showed that SHMT2 silencing remarkably reduced the healing area of HN6 and HSC3 cells (Fig. 6g).

Then we injected HN6 cells subcutaneously into the left inner thighs of NOD/SCID mice and observed the growth of the subsequent tumors. As can be seen in Fig. 6h, the volumes and weights of tumors in mice in the sh-SHMT2 cell injection group were significantly smaller than those in mice of the control group. After sacrificing the mice, we extracted RNA from the tumors and measured the mRNA expression of *SHMT2* and cell cycle regulators. The *SHMT2* expression level was significantly reduced in tumors from the sh-SHMT2 mice group compared with that from the control group. The mRNA levels of *CDKN1A* and *CDKN1B* were increased, whereas *CCDN1* and *CDK4* were decreased in the sh-SHMT2 mice (Fig. 6i). Immunohistochemistry analysis of tumors from mice showed that SHMT2 and Ki-67 protein expression levels were downregulated in the sh-SHTM2 group (Fig. 6j). P21^Cip1^ and P27^Kip1^ protein expression levels were up-regulated in the sh-SHMT2 group. CyclinD1 and CDK4 protein expression levels, however, were downregulated in the sh-SHMT2 group compared with the control group (Fig. 6k). These findings supported the hypothesis that SHMT2 deletion inhibited TSCC cell proliferation and tumor growth in vivo.

## Discussion

One-carbon metabolism provides cellular components such as nucleotides, lipids, and proteins for cell growth, and its role in tumorigenesis has attracted considerable attention in recent years [23–25]. SHMT2 is a mitochondrial enzyme in one-carbon metabolism that promotes the conversion of serine and 5,10-tetrahydrofolate (THF) to glycine and methylene-THF (me-THF). Serine and glycine are considered to be necessary for cell proliferation[26–28]. Moreover, a methyl group produced in one-carbon metabolism is required for histone, DNA and RNA methylation, which are essential for DNA damage repair and DNA stability[28, 29]. Several studies have validated that SHMT2 expression is elevated in breast cancer, intrahepatic cholangiocarcinoma and gastric cancer, and high levels of SHMT2 are associated with poor prognosis of patients[15, 30, 31]. Study in gliomas found that SHMT2 contributes to cancer cell survival in the harsh tumor microenvironment, which relies on glycine clearance [18]. Another study in breast cancer demonstrated that SHMT2 acts as the first rate-limiting enzyme in one-carbon pathway, promoting rapid cell growth and increasing the potential for metastasis [32]. These findings suggested that abnormal expression of SHMT2 protein exerts a profound influence on tumors progression.

In the current study, we found that SHMT2 was overexpressed in OSCC tissues through analysis of TCGA database samples, which was confirmed by experiments including immunohistochemical staining using clinic OSCC tissues, western blot and RT-qPCR analysis of TSCC cell lines. Further analysis with TCGA data found that highly-expressed SHMT2 was remarkably correlated with unfavorable overall survival, disease-specific survival and disease-free survival. Moreover, clinical significance analysis revealed that high expression of SHMT2 was significantly correlated with advanced pathologic tumor stage, lymph node metastasis and advanced clinic stage. Although there was no statistical significance between SHMT2 expression and histologic grade, lymph node neck dissection, lymph vascular invasion and perineural invasion, we speculated that it might be caused by the limited sample size. These findings were in accordance with studies in hepatocellular carcinoma and gastric cancer[16, 31], which suggested that SHMT2 could serve as an indictive biomarker for prognosis of TSCC.

In gliomas, overexpression of SHMT2 was found to enhance the proliferation and invasion of tumor cells[11]. Additionally, Yang and his colleagues proved that down regulating of SHMT2 suppresses the proliferation of the colon colorectal carcinoma cells and hinders the tumor cell growth in vitro and vivo [14]. However, the underlying specific mechanism remains unclear. On the purpose of exploring the role of SHMT2 in OSCC, we conducted WGCNA with OSCC samples selected from TCGA database to screen the gene sets related with SHTMT2 expression. And the most significant module related with SHMT2 expression was identified by WGCNA, consisting of 159 genes. Following the results of GO and KEGG analysis, the hub module genes were principally enriched in nuclear division and cell cycle. Notably, one study has reported that silencing the SHMT2 gene and depleting extracellular glycine prolonged the G1 phase of the cell cycle, thereby slowing down the proliferation of HeLa cells [27]. me-THF, formed by SHMT2 catalyzation, is then transferred to 10-formyltetrahydrofolate(F-THF), which plays a vital role in purine synthesis [33]. Moreover, the GO and KEGG analysis showed that the influence of SHMT2 on proliferation of OSCC was significantly linked with cell cycle regulation. For GSEA, we found that SHMT2 high-expression group was more remarkably correlated with cell cycle, cell cycle checkpoint, positive regulation of cell cycle transition and cell cycle G1/S transition signal pathways when compared with SHMT2 low-expression group. These findings cooperated to confirm that SHMT2 play a noticeable role in OSCC cell cycle regulation.

To validate the findings of bioinformatic analysis, we further measured the proliferation of HN6 and HSC3 cells after silencing SHMT2 by CCK-8 and Edu staining. Consistently, inhibition of SHMT2 attenuated cell viability and proliferation capacity of TSCC cells. In addition, flow cytometric analysis and western blot were performed to evaluate the changes in cell cycle phase and cell cycle related protein expression levels. Similarly, low expression of SHMT2 prolonged the G1 phase of cell cycle, causing more cells to stagnate in G1 phase compared with control group. Western blot analysis showed that p21^Cip1^ and p27^Kip1^ protein level elevated, while cyclinD1 and CDK4 protein expression reduced by SHMT2 knockdown. Research in lung cancer has indicated that the cell cycle can be arrested owing to a decrease in the expression level of SHMT1 [34]. In comparison, our study suggested that silencing SHMT2 in TSCC cells triggered changes in cell cycle regulators and induced cells block in G1 phase of the cell cycle, which may be related to decreased cell proliferation. Likewise, silencing SHMT2 effectively impeded the tumor growth in vivo. However, whether the cell cycle changes induced by abnormal SHMT2 expression are sufficient to inhibit the growth of TSCC cells remains to be further studied.

Epithelial mesenchymal transition (EMT) plays an important role in tumor progression, and is known as an important mechanism to facilitate tumor migration and invasion [35, 36]. SHMT2 was found to be related with the production of NADPH [37]. Modulation of the NADPH/NADP^+^ ratio is directly linked with redox balance during hypoxia [38]. The degree of hypoxia is positively linked with metastasis and hypoxia-inducible factor1α (HIF-1α) acts as a prerequisite role in EMT of tumors [39]. It’s reasonable to speculate that there may be some potential association between SHMT2 and EMT of tumors. Moreover, HIF-1α was validated to be able to induce SHMT2 expression in hypoxic microenvironment [40]. Albert M. Li et al. [32] found that SHMT2 expression increases in breast invasive carcinoma. In vitro, SHMT2 knockdown was more effectively inhibited proliferation in these highly metastatic subclones than in parental population. In vivo, SHMT2 inhibition also reduced breast cancer growth of primary and metastatic sites. In our study, we detected the invasive and migrative ability of TSCC cells after treated with si-RNA and sh-RNA through trans-well assays. Meanwhile, EMT-related protein expression levels were examined by western blot. It’s found that knockdown of SHMT2 significantly suppressed the TSCC cells invasion and migration in vitro. These findings were consistent with results in breast cancer and hepatocellular carcinoma [41, 42].

Recently, SHMT2 has been reported as a target for cancer chemotherapy. Pemetrexed has been used as an antifolate drug targeting SHMT2 in vitro [43]. Leucovorin (N5CHO-THF) and 3-bromopyruvate (3BP) are two selective inhibitors for SHMT2 that have been examined in clinical trials [44, 45]. These work suggested that SHMT2 may be a potential therapeutic target for cancer patients.

This study focused on the influence of SHMT2 in TSCC progression, aiming to provide some new insights into the role of SHMT2 in TSCC treatment. However, there are some limitations in our research, such as the lack of direct evidence clarifying the association between cell cycle arrest and cell proliferation, which provides clues for our further efforts to explore the exact mechanism of SHMT2 regulating TSCC cell proliferation (Additional file 2).

In summary, despite the above limitations, our research demonstrated that SHMT2 was up-regulated in oral cancer tissues and was associated with poor prognosis of oral cancer patients. Moreover, silencing of SHMT2 significantly inhibited cell proliferation, cell cycle, invasion and migration of TSCC cells. All these findings provided new understanding about aggressive progression of TSCC and might be beneficial to find a better treatment option for TSCC patients (Additional files 3 and 4).

## Supplementary Information

**Additional file 1: Fig. S1. **WGCNA for OSCC samples in GSE30784. (a) Hierarchical clustering dendrogram of 164 samples in GSE30784. (b) Analysis of the scale-free fit index and the mean connectivity for various soft threshold power. (c) The scale free topology test based on β = 5. (d) The cluster dendrogram of gene modules with dissimilarity based on topological overlap. (e) The cluster dendrogram of module eigengenes: modules below the red line (0.2) are merged. (f) Correlation coefficient and *P*-value for module-trait relationship. **Fig. S2. **Testification of SHMT2 related pathways using samples in GSE30784 via GO, KEGG and GSEA.**. **(a) Biological process analysis for the black module. (b) KEGG enrichment analysis for the black module. (c–h) GSEA enrichment analysis: GO and KEGG pathway genes in SHMT2 high versus low expression using samples in GSE30784. ES, enrichment score.

**Additional file 2.** Information about cell sources and table revisions

**Additional file 3.** Genes enriched in tan module

**Additional file 4.** Genes enriched in black module

## Data Availability

The datasets used in this study can be obtained from the Cancer Genome Atlas (TCGA) database (https://www.cancer.gov/tcga) and the Gene Expression Omnibus (GEO) database (https://www.ncbi.nlm.nih.gov/).

## Abbreviations

ES: Enrichment score
FBS: Fetal bovine serum
ANCT: Adjacent noncancerous tissue samples
GO: Gene Ontology
GSEA: Gene set enrichment analysis
KEGG: Kyoto Encyclopedia of Genes and Genomes
OSCC: Oral squamous cell carcinoma
PBS: Phosphate-buffered saline
TCGA: The Cancer Genome Atlas
TOM: Topological overlap matrix
WGCNA: Weighted gene co-expressed network analysis
