# Supplementary material for: Silencing SHMT2 inhibits the progression of tongue squamous cell carcinoma through cell cycle regulation
Source: Cancer Cell Int. 2021 Apr 16;21:220. doi: 10.1186/s12935-021-01880-5 (PMC8052717; doi:10.1186/s12935-021-01880-5)
Supplement: Supplementary file 1 — Additional file 1: Fig. S1. WGCNA for OSCC samples in GSE30784. (a) Hierarchical clustering dendrogram of 164 samples in GSE30784. (b) Analysis of the scale-free fit index and the mean connectivity for various soft threshold power. (c) The scale free topology test based on β = 5. (d) The cluster dendrogram of gene modules with dissimilarity based on topological overlap. (e) The cluster dendrogram of module eigengenes: modules below the red line (0.2) are merged. (f) Correlation coefficient and P-value for module-trait relationship. Fig. S2. Testification of SHMT2 related pathways using samples in GSE30784 via GO, KEGG and GSEA.. (a) Biological process analysis for the black module. (b) KEGG enrichment analysis for the black module. (c–h) GSEA enrichment analysis: GO and KEGG pathway genes in SHMT2 high versus low expression using samples in GSE30784. ES, enrichment score. [file 12935_2021_1880_MOESM1_ESM.docx]

**Supplementary material**

**Identification of co-expression networks in GSE30784**

We conducted WGCNA for GSE30784 to validate the results of OSCC samples in TCGA database. The top 50% of 11674 genes and 164 OSCC samples were applied to construct gene co-expression network (Fig. S1a). Soft-thresholding power was set at 5 (scale free R^2^ = 0.85) to obtain an appropriate scale free topology index (Fig. S1b, c). As a result, 31 co-expression modules were constructed and 29 highly similar modules were merged based on similarity coefficient higher than 0.8 (Fig. S1d, e). The black module (*r*=0.43, *P*<0.0001) was finally identified the most related with SHMT2 expression (Fig. S1e).


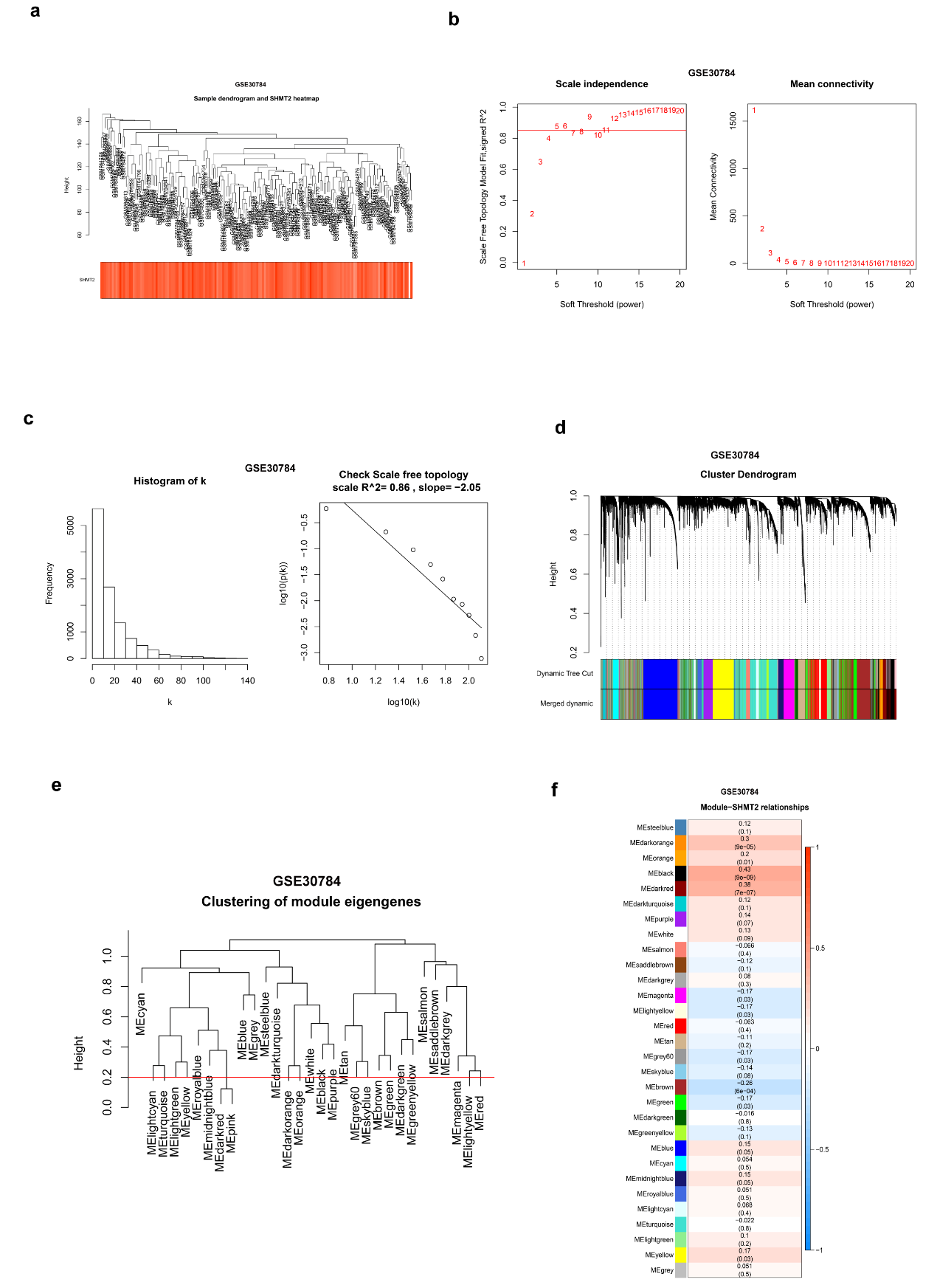


**Fig. S1** WGCNA for OSCC samples in GSE30784

(a) Hierarchical clustering dendrogram of 164 samples in GSE30784

(b) Analysis of the scale-free fit index and the mean connectivity for various soft threshold power.

(c) The scale free topology test based on β=5.

(d) The cluster dendrogram of gene modules with dissimilarity based on topological overlap.

(e) The cluster dendrogram of module eigengenes: modules below the red line (0.2) are merged.

(f) Correlation coefficient and *P*-value for module-trait relationship.

**Go, KEGG and GSEA analysis for black module**

We also performed the GO and KEGG enrichment analysis for the black module to verify the biological function of SHMT2. GO analysis indicated that biological process of the black module was mainly involved in the nuclear division, chromosome segregation and DNA replication (*P*<0.0001) (Fig. S2a). KEGG analysis also showed that genes were most significantly enriched in cell cycle (*P*<0.001) (Fig. S3b). And these results were in accordance with analysis with TCGA data.

As results of Gene set enrichment analysis (GSEA) shown, overexpressed SHMT2 was positively associated with positive regulation of cell cycle transition, cell cycle G1/S phase transition, cell cycle check point and cell cycle DNA replication (*P*<0.0001) (Fig. S3c-h).

**
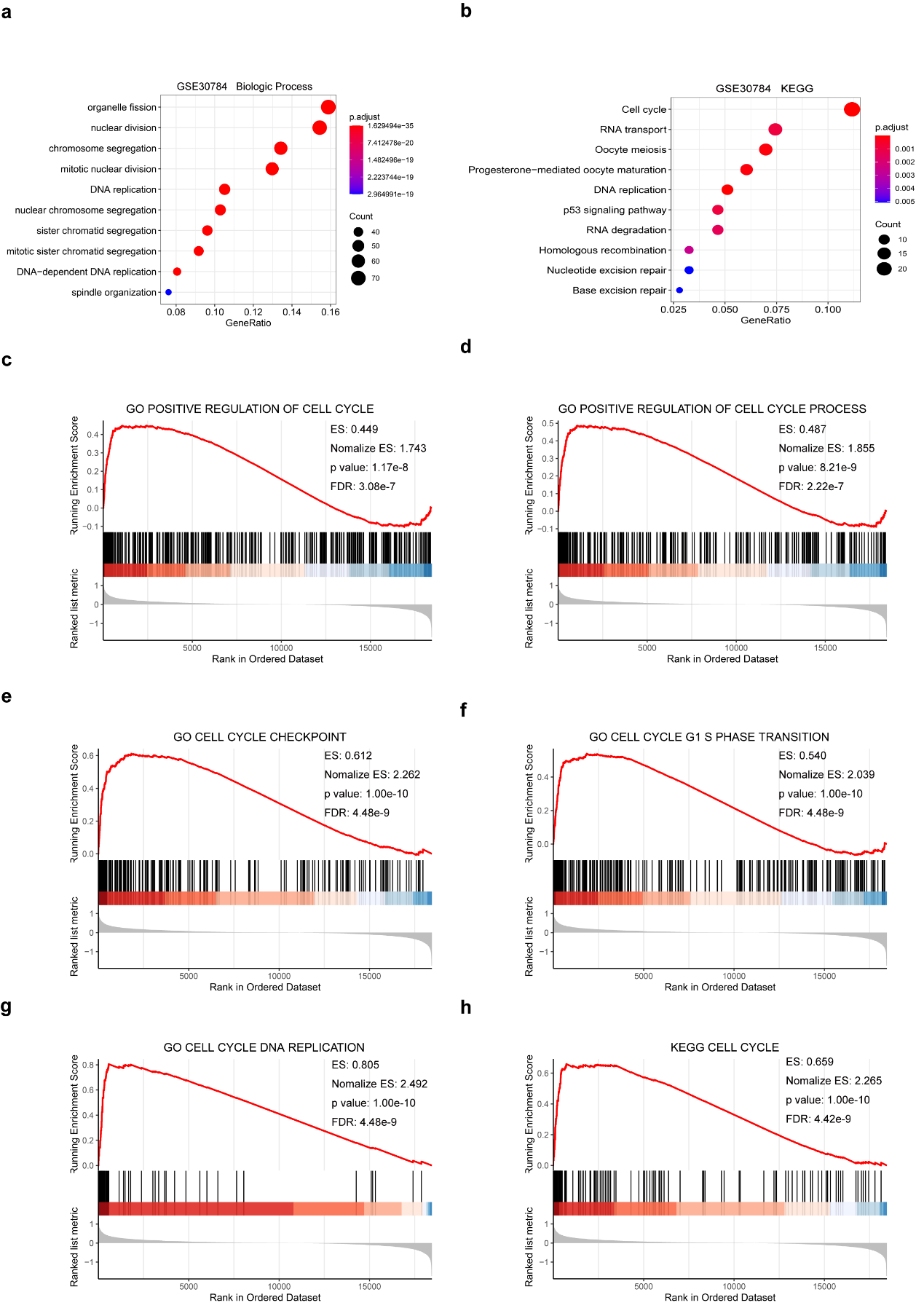
**

**Fig. S2** Testification of SHMT2 related pathways using samples in GSE30784 via GO, KEGG and GSEA.

(a) Biological process analysis for the black module.

(b) KEGG enrichment analysis for the black module.

(c-h) GSEA enrichment analysis: GO and KEGG pathway genes in SHMT2 high versus low samples in GSE30784. ES, enrichment score.
