# Supplementary material for: Silencing SHMT2 inhibits the progression of tongue squamous cell carcinoma through cell cycle regulation
Source: Cancer Cell Int. 2021 Apr 16;21:220. doi: 10.1186/s12935-021-01880-5 (PMC8052717; doi:10.1186/s12935-021-01880-5)
Supplement: Supplementary file 2 — Additional file 2. Information about cell sources and table revisions [file 12935_2021_1880_MOESM2_ESM.docx]

**The detailed information of OSCC cell lines in this paper**

| No. | Cell type | JCRB No. | Cell Name | Organism | Tissue | Profile |
| --- | --- | --- | --- | --- | --- | --- |
| 1 | general cells | JCRB0623 | HSC-3 | Homo sapiens | tongue | Human oral squamous carcinoma cell line with high metastatic potential. |
| No. | Cell type | DSMZ No. | Cell Name | Organism | Tissue | Profile |
| 2 | general cells | **ACC 447** | CAL-33 | Homo sapiens | tongue | Tongue squamous cell carcinoma |
| No. | Cell type | ATCC No. | Cell Name | Organism | Tissue | Profile |
| 3 | general cells | **ATCC®CRL-1623** | SCC15 | Homo sapiens | tongue | squamous cell carcinoma |
| 4 | general cells | **ATCC® CRL-1628** | SCC25 | Homo sapiens | tongue | squamous cell carcinoma |
| 5 | general cells | **Provided by Professor Liu (Southern Medical University, Guangdong, China)** | HN6 | Homo  sapiens | tongue | squamous cell carcinoma |
| 6 | general cells | **provided by J. Silvio Gutkind (NIH, Bethesda, MD, USA)** | NOK | Homo  sapiens | Oral mucosa | normal oral keratinocyte |

**The original Table 1**

**Table1 Correlations between SHMT2 and clinicopathological characteristics of oral squamous cell carcinoma in TCGA database（n=103）**

|  | **SHMT2 expression** | | | | |
| --- | --- | --- | --- | --- | --- |
| **Characteristics** | **High** | **Low** | | ***p* value** | |
| Anatomic site |  |  | |  | |
| Alveolar Ridge | 1 | 1 | | 0.321 | |
| Buccal Mucosa | 20 | 10 | |  | |
| Floor of mouth | 15 | 9 | |  | |
| Oral Cavity | 17 | 2 | |  | |
| Tongue | 20 | 8 | |  | |
| Age (years) | |  | | | |
| < 61 | 25 | 30 | **0.031^*^** | | |
| ≥ 61 | 32 | 16 |  |  |  |
| Gender |  |  |  | |  |
| Male | 39 | 32 | 0.901 | | |
| Female | 18 | 14 |  |  |  |
| Alcohol history | |  |  | |  |
| Yes | 23 | 7 | **0.005^**^** | | |
| No | 34 | 39 |  |  |  |
| Tobacco smoking history | | |  | |  |
| Yes | 19 | 12 | 0.425 | | |
| No | 38 | 34 |  |  |  |
| T stage |  |  |  | |  |
| T1-2 | 16 | 22 | **0.04^*^** | | |
| T3-4 | 41 | 24 |  |  |  |
| N stage |  |  |  | |  |
| N^-^ | 17 | 21 | 0.098 | | |
| N^+^ | 40 | 25 |  |  |  |
| M stage |  |  |  | |  |
| M^-^ | 42 | 38 | 0.280 | | |
| M^+^ | 15 | 8 |  |  |  |
| Clinical stage | |  |  | |  |
| I-II | 5 | 14 | **0.005^**^** | | |
| III-IV | 52 | 32 |  |  |  |
| Histologic grade | |  |  | |  |
| Well | 4 | 12 | **0.028^*^** | | |
| Moderately | 40 | 27 |  |  |  |
| Poorly | 13 | 7 |  |  |  |
| Lymph node neck dissection | | |  | |  |
| Yes | 3 | 3 | 0.554 | | |
| No | 54 | 43 |  |  |  |
| Lymph vascular invasion | | |  | |  |
| Yes | 36 | 34 | 0.245 | | |
| No | 21 | 12 |  |  |  |
| Perineural invasion | |  |  | |  |
| Yes | 31 | 35 | **0.022^*^** | | |
| No | 26 | 11 |  |  |  |
| Close or positive margin | | |  | |  |
| Yes | 34 | 35 | 0.078 | | |
| No | 23 | 11 |  |  |  |
| Recurrence | |  |  | |  |
| Yes | 35 | 36 | 0.066 | | |
| No | 22 | 10 |  |  |  |

*represents *P* < 0.05, **represents *P*<0.01, ***represents *P*<0.001

**The revised Table1 (revised portion was highlighted in yellow)**

**Table1 Correlations between SHMT2 and clinicopathological characteristics of oral squamous cell carcinoma in TCGA database（n=96）**

|  | **SHMT2 expression** | | | | |
| --- | --- | --- | --- | --- | --- |
| **Characteristics** | **High** | **Low** | | ***p* value** | |
| Anatomic site |  |  | |  | |
| Alveolar Ridge | 5 | 5 | | 0.215 | |
| Buccal Mucosa | 6 | 3 | |  | |
| Floor of mouth  Hard palate | 20  0 | 9  2 | |  | |
| Oral Cavity | 11 | 7 | |  | |
| Tongue | 12 | 16 | |  | |
| Age (years) | |  | | | |
| < 61 | 24 | 28 | **^0.03*^** | | |
| ≥ 61 | 30 | 14 |  |  |  |
| Gender |  |  |  | |  |
| Male | 36 | 30 | 0.618 | | |
| Female | 18 | 12 |  |  |  |
| Alcohol history | |  |  | |  |
| Yes | 32 | 36 | **0.005**** | | |
| No | 22 | 6 |  |  |  |
| Tobacco smoking history | | |  | |  |
| Yes | 36 | 30 | 0.618 | | |
| No | 18 | 12 |  |  |  |
| pT stage |  |  |  | |  |
| T1-2 | 13 | 20 | **^0.016*^** | | |
| T3-4 | 41 | 22 |  |  |  |
| pN stage |  |  |  | |  |
| N^-^ | 17 | 20 | **0.039*** | | |
| N^+^ | 37 | 22 |  |  |  |
| pM stage |  |  |  | |  |
| M^-^ | 39 | 34 | 0.320 | | |
| M^+^ | 15 | 8 |  |  |  |
| Clinical stage | |  |  | |  |
| I-II | 8 | 17 | **0.004**** | | |
| III-IV | 46 | 25 |  |  |  |
| Histologic grade | |  |  | |  |
| Well | 4 | 10 | 0.078 | | |
| Moderately | 39 | 25 |  |  |  |
| Poorly | 11 | 7 |  |  |  |
| Lymph node neck dissection | | |  | |  |
| Yes | 52 | 40 | 1.000 | | |
| No | 2 | 2 |  |  |  |
| Lymph vascular invasion | | |  | |  |
| Yes | 20 | 10 | 0.165 | | |
| No | 34 | 32 |  |  |  |
| Perineural invasion | |  |  | |  |
| Yes | 26 | 23 | ^0.520^ | | |
| No | 28 | 19 |  |  |  |
| Close or positive margin | | |  | |  |
| Yes | 22 | 9 | **0.045*** | | |
| No | 32 | 33 |  |  |  |
| Recurrence | |  |  | |  |
| Yes | 21 | 10 | 0.117 | | |
| No | 33 | 32 |  |  |  |

pT stage: pathologic T stage

pN stage: pathologic N stage

pM stage: pathologic M stage

*represents *P* < 0.05, **represents *P*<0.01, ***represents *P*<0.001

**Original Table2**

**Table2 Univariate and multivariate analysis of various clinicopathologic characteristics related with overall survival in OSCC patients (n=303)**

|  | | | | **Univariate analysis** | | | | **Multivariate analysis** | | |
| --- | --- | --- | --- | --- | --- | --- | --- | --- | --- | --- |
|  | | | | **HR (95% CI)** | | ***P*** | **HR (95% CI)** | | ***P*** |  |
| Gender | | 1.062(0.753~1.497) | | | | 0.733 | | 0.95(0.661~1.367) | 0.783 | |
| Age | | 1.203(0.862~1.679) | | | | 0.277 | | 1.201(0.846~1.706) | 0.305 | |
| T stage | | 1.329(0.94~1.879) | | | | 0.108 | | 1.5(0.783~2.875) | 0.222 | |
| N stage | | 1.453(1.046~2.017) | | | | **0.026^*^** | | 1.51(0.991~2.299) | 0.055 | |
| M stage | | 0.697(0.172~2.821) | | | | 0.613 | | 0.686(0.168~2.809) | 0.601 | |
| Clinical stage | | 1.329(0.914~1.933) | | | | 0.136 | | 0.696(0.313~1.548) | 0.374 | |
| Histological grade | | 1.286(0.992~1.667) | | | | 0.058 | | 1.32(1.012~1.72) | **0.04^*^** | |
| SHMT2 level | | | 1.548(1.111~2.156) | | | | **0.01^*^** | 1.478(1.051~2.078) | **0.025^*^** | |

*represents *P* < 0.05, **represents *P*<0.01, ***represents *P*<0.001

**Revised Table2 (revised portion was highlighted in yellow)**

**Table2 Univariate and multivariate analysis of various clinicopathologic characteristics related with overall survival in OSCC patients (n=303)**

|  | | | | **Univariate analysis** | | | | **Multivariate analysis** | | |
| --- | --- | --- | --- | --- | --- | --- | --- | --- | --- | --- |
|  | | | | **HR (95% CI)** | | ***P*** | **HR (95% CI)** | | ***P*** |  |
| Gender | | 0.924(0.656~1.300) | | | | 0.648 | |  |  |  |
| Age | | 1.185(0.853~1.646) | | | | 0.311 | |  |  |  |
| cT stage | | 1.344(0.951~1.899) | | | | 0.094 | |  |  |  |
| cN stage | | 1.471(1.060~2.039) | | | | **0.021^*^** | | 1.534(1.008~2.335) | **0.046^*^** | |
| cM stage | | 0.692(0.171~2.799) | | | | 0.605 | |  |  |  |
| Clinical stage | | 1.342(0.923~1.950) | | | | 0.123 | |  |  |  |
| Histological grade | | 1.282(0.990~1.661) | | | | 0.06 | | 1.317(1.011~1.715) | **0.041^*^** | |
| SHMT2 level | | | 1.522(1.094~2.117) | | | | **0.013^*^** | 1.452(1.034~2.037) | **0.031^*^** | |

cT stage: clinic T stage

cN stage: clinic N stage

cM stage: clinic M stage

*represents *P* < 0.05, **represents *P*<0.01, ***represents *P*<0.001
